# Mitochondrial Fission Protein 1: Emerging Roles in Organellar Form and Function in Health and Disease

**DOI:** 10.3389/fendo.2021.660095

**Published:** 2021-03-25

**Authors:** Ugochukwu Kelvin Ihenacho, Kelsey A. Meacham, Megan Cleland Harwig, Michael E. Widlansky, R. Blake Hill

**Affiliations:** ^1^ Department of Biochemistry, Medical College of Wisconsin, Milwaukee, WI, United States; ^2^ Department of Medicine, Division of Cardiovascular Medicine, Medical College of Wisconsin, Milwaukee, WI, United States

**Keywords:** mitochondria, mitophagy, FIS1, apoptosis, mitochondrial dynamics, diabetes, cancer, neurodegenerative diseases

## Abstract

Mitochondrial fission protein 1 (Fis1) was identified in yeast as being essential for mitochondrial division or fission and subsequently determined to mediate human mitochondrial and peroxisomal fission. Yet, its exact functions in humans, especially in regard to mitochondrial fission, remains an enigma as genetic deletion of Fis1 elongates mitochondria in some cell types, but not others. Fis1 has also been identified as an important component of apoptotic and mitophagic pathways suggesting the protein may have multiple, essential roles. This review presents current perspectives on the emerging functions of Fis1 and their implications in human health and diseases, with an emphasis on Fis1’s role in both endocrine and neurological disorders.

## Introduction

Popularly regarded as the cell’s “powerhouse”, mitochondria are found in nearly every human tissue where they are sculpted for numerous essential processes. In addition to ATP generation, mitochondria are the sole centers of iron-sulfur cluster biosynthesis, where heme biosynthesis begins, and centers of intermediary metabolism and signaling. Apoptosis is regulated at the mitochondrion and epigenetic regulation of gene transcription relies on mitochondrial-derived metabolites. First observed ubiquitously in fixed tissues as refractive granules termed “bioblasts” by Richard Altmann in 1890 (1), these subcellular organelles were called by many names until convergence on the term mitochondria – aptly coined by Carl Benda in 1899 to reflect their diverse morphology using the Greek words for thread (mitos) and granule (chondrion). This diverse morphology is not static as mitochondria were recognized to undergo frequent fission/division and fusion/union events over a hundred years ago (2–4), however, the functional relevance was unclear until recently when the fission and fusion genes were discovered and found to be mutated in human disease [For excellent reviews see here (5–8)]. This work, largely in the last 20 years, has defined the field of mitochondrial dynamics which is also related to mitochondrial motility, removal (mitophagy), biogenesis, and apoptosis in ways that are still being defined. Proper mitochondrial networks are clearly critical hubs for cell division and managing cellular stress through mitophagic and apoptotic signaling, which appears to involve unopposed mitochondrial fission. Given this importance, it is perhaps not surprising that the fission/fusion machinery is frequently dysregulated in human diseases.

A central question that has persisted since the identification of mitochondria is the relationship between form and function (9). Mitochondrial form, or morphology, results from the net actions of complex dynamin GTPases that control mitochondrial fusion-fission. In mammals, fusion of apposing mitochondrial outer membranes is mediated by integral membrane proteins Mfn1 and 2, while OPA1 mediates inner membrane fusion and cristae remodeling (10–14). Outer membrane fission is mediated predominantly by a dynamin superfamily GTPase, Drp1, that is localized to the cytoplasm and somehow recruited to scission sites by resident outer-membrane proteins including Mff, Fis1, Mid49 and Mid51 (15–25). Despite their bacterial origins, metazoan mitochondria do not share the bacterial division machinery where scission is accomplished by the tubulin ortholog, FtsZ, which is a GTPase that assembles at the cell midpoint on the inside of the membrane. From phylogenetic analysis, it appears that most eukaryotes share FtsZ orthologs, whereas metazoan mitochondria do not (26). To date, no inner membrane fission machinery has been identified, although recent evidence suggests that OPA1 and MTP18 may be involved (27–29). What determines a site of fusion or fission is unknown, but appears to involve inter-organelle communication with the endoplasmic reticulum (30–32) and also other organelles (33, 34). ER-mitochondria contact sites (ERMCs) are microdomains where both organelle membranes stay juxtaposed, presumably for exchange of lipids, calcium, and other metabolites. Sites of fission may also be coordinated with localization of mtDNA nucleoids that reside on the matrix side of the inner membrane, which can allow segregation of healthy from damaged organelles (35, 36).

Defining the mitochondrial form/function relationship is a challenge in part because mitochondria adopt a wide variety of tissue-specific morphologies. For instance, mitochondria in heart muscles (cardiomyocytes) are highly ordered and elongated tubules, whereas in liver (hepatocytes) are punctiform and dispersed. This suggests that mitochondrial connectivity is dispensable in some cell types. Nevertheless, a degree of balance between mitochondrial fusion and fission is necessary for the maintenance of bioenergetic function (37–40). While the mitochondrial proteome is sculpted in a tissue-specific manner to accommodate distinct energetic and metabolic needs (41, 42), how the mitochondrial network accommodates this is unclear. A challenge for the field in the future is to define the exact form/function relationship, which will greatly benefit from new computational approaches that quantitatively describe the mitochondrial form/network (43–53). The form/function relationship is also challenged by mitochondrial morphological changes during motility, mitophagy and apoptosis. Mitochondrial motility involves the trafficking of mitochondria along cytoskeletal elements. Central to this is outer-membrane Rho GTPases, RHOT1/Miro1 and RHOT2/Miro2, that mobilize mitochondria along actin and microtubule tracks by virtue of their calcium sensing domains (54–59). Lastly, mitochondrial elimination through proteostasis (60, 61), autophagy, apoptosis and other mechanisms (62, 63) is carried out by molecular cascades involving a complex array of proteins from multiple cellular compartments which can vary by physiological context (64–67).

This review focuses on only one of the fission genes, FIS1, with the rationale of examining current perspectives and controversies regarding its physiological role in fission-fusion, mitophagy, and apoptosis in relation to human health and disease. The main controversy surrounding Fis1 is what is its primary function in the cell. A collective body of evidence indicates Fis1 plays a direct role in mitochondrial fission, however, as discussed in detail below, more recent evidence suggests a prominent role in mitophagy. Its role in apoptosis is widely acknowledged but the exact mechanistic details are unclear. Here, we will examine the current evidence for, and against, a role for Fis1 in these three processes. For other aspects of mitochondrial dynamics, we refer the interested reader to these outstanding reviews (5, 6, 54, 68, 69).

## Evolution of the Rudimentary Fission Machinery From Simple to Higher Eukaryotes

Fis1 was discovered by complementation screens in *Saccharomyces cerevisiae* designed to identify genes that could rescue temperature-sensitive alleles of either of the fusion genes, Fzo1 (64, 70) or Mgm1 (71) that failed to grow on non-fermentable glycerol at non-permissive temperatures due to unopposed fission and subsequent loss of mitochondrial DNA (70, 72–74). Using non-fermentable sugar sources requires yeast to use mitochondrial oxidative phosphorylation, which is impaired upon loss of mtDNA. Thus the screen identified genes that could prevent runaway fission and subsequent mtDNA loss. In addition to the mitochondrial dynamin Dnm1, the screen identified two more genes now called Fis1 and Mdv1. Mutations in these genes rescued mitochondria from fragmenting without affecting mitochondrial fusion, making them legitimate fission proteins. Human orthologs of Fis1 (9, 10, 75) and Dnm1 (76, 77), but not Mdv1, have been identified. We note that in this review we do not equate fragmentation with fission, but rather use fragmentation to refer to the morphological observation of the mitochondrial network that can arise from either activated fission or impaired fusion.

**Figure 1:**
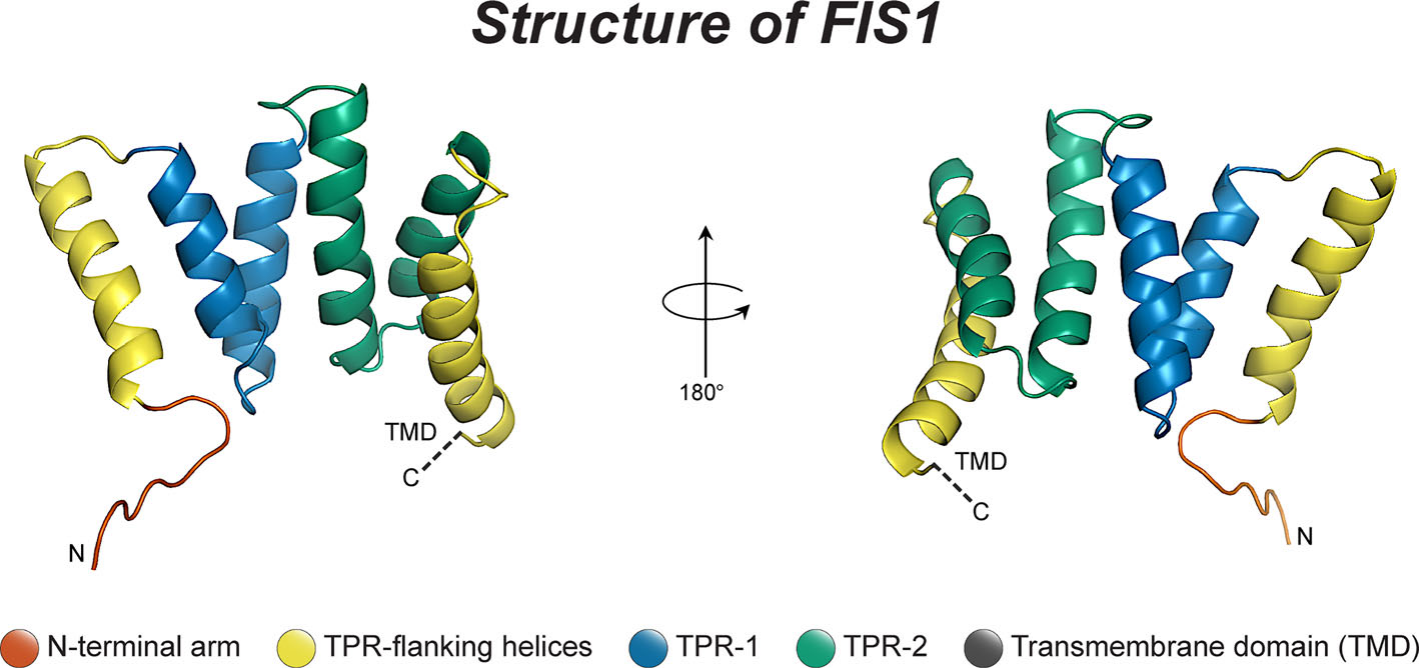
The structure of human Fis1. The structure of human Fis1 (NMR structure PDB: 1PC2). Fis1 is anchored to the mitochondrial outer membrane by a C-terminal transmembrane domain exposing a soluble domain to the cytoplasm that is comprised of two core tetratricopeptide repeat (TPR) structural motifs flanked by two helices (helices 1 and 6). The N-terminal region of Fis1 is referred to as the “N-terminal arm”. The transmembrane domain (TMD) has been truncated here and its structure in the membrane is unknown but presumably helical.

**Figure 2:**
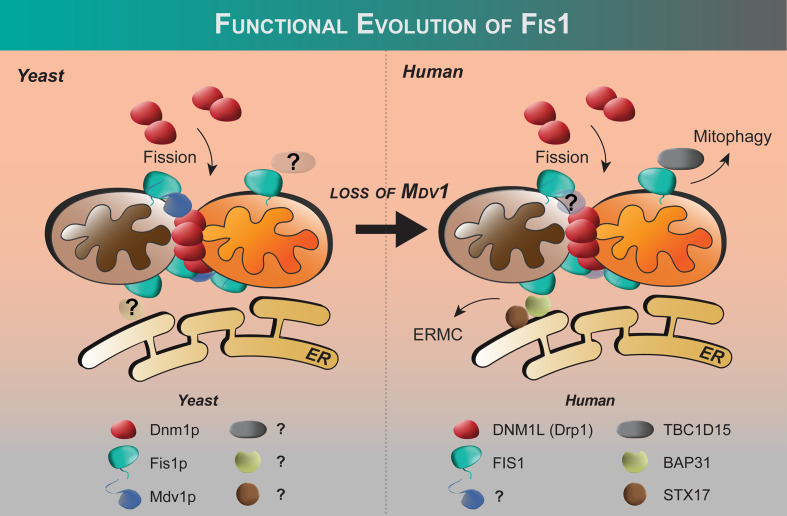
Functional evolution of Fis1 from yeast to humans. Fis1 interacting partners appear to differ between fungi and higher eukaryotes. In budding yeast, the fission mechanoenzyme Dnm1p is recruited to mitochondria by resident mitochondrial protein Fis1p with Mdv1p acting as an adapter. In yeast, Fis1p appears to play an early and late role in Dnm1p assembly (86). No human ortholog of Mdv1p has been identified to date, and human Fis1 is thought to recruit proteins other than the fission mechanoenzyme Drp1 to the mitochondrion (see text). The extent of the Fis1 interactome is not fully defined.

Fis1 is a C-terminally tail-anchored protein in the mitochondrial outer membrane that exposes a 15kDa soluble domain (**Figure 1**) to the cytoplasm (16, 17, 78). This soluble domain adopts two tetratricopeptide repeats, or TPRs (75, 79), which are common protein-protein interaction domains (75, 79–82). Consistent with its structure, yeast Fis1p recruits an adapter, Mdv1p, and the fission mechanoenzyme, Dnm1p, to sites of scission (73, 74, 83–85). In accordance with this model (**Figure 2**), Mdv1p and Dnm1p mitochondrial localization was dependent on Fis1p, and genetic ablation of *fis1* could sufficiently rescue Fzo1 loss of function (24, 73). This model was supported by subsequent structural studies which showed that Mdv1p (and paralog Caf4p) facilitated Fis1p-Dnm1p interaction by acting as an adapter (87, 88). Curiously, Fis1p is uniformly localized on the mitochondrial surface, but fission occurs at in discrete sites. Thus, the necessity for Fis1p in mitochondrial-dynamin mediated fission is not disputed, although its exact role in assembling the fission machinery and determining a site of scission remains to be elucidated, but may involve dimerization (89). A clue to this process is mutation of a conserved Fis1p residue that weakens its affinity for Mdv1p and causes Dnm1p to colocalize uniformly on the mitochondrial surface, indicating that functional Fis1p is necessary for Dnm1p assembly into pre-scission puncta (86).

Shortly after the discovery of Fis1 in yeast, orthologs in higher eukaryotes were discovered including in human cells (16, 17, 78). Fis1 overexpression causes mitochondrial fragmentation that is eliminated upon co-expression with a dominant negative mutation of Drp1 (K38A) (16, 17, 78). Fis1 knockdown by siRNA causes mitochondrial elongation and delayed apoptosis (**Table 1**) **(** 97, 99, 100). These findings were somewhat surprising given that no orthologs of Mdv1p/Caf4p have been identified in mammals, nor outside the fungi kingdom. Nevertheless, these seminal studies showed that Fis1 in humans is an evolutionarily conserved recruiter of Drp1, consistent with their similar structures despite sharing only 26% sequence identity (67% similarity). Fis1 and Drp1 are also localized to peroxisomes for fission (93, 94).

**Table 1:** The effects of Fis1 overexpression or knock-out/down on mitochondrial morphology.

Cell lines	Fis1 Overexpression	Fis1 KD/KO	References
*143B-ρ0 Cybrid B4*	*Increased mitochondrial fragmentation; decreased translocation of GLUT1 and GLUT4; increased ROS production.*	*KD; Increased mitochondrial elongation; increased translocation of GLUT1 and GLUT4; decreased ROS production.*	(90)
*ALS patient derived fibroblasts*	*N/A*	*KD; increased mitochondrial elongation.*	(91)
*BHK-21*	*Increased mitochondrial fragmentation.*	*N/A*	(80)
*Clone 9*	*Increased mitochondrial fragmentation.*	KD; increased mitochondrial elongation (16).	(16, 80)
*Chang; Mv1lu*	*Inhibition of DFO-induced mitochondrial elongation and cell size increase; Partial inhibition of S-Bgal expression.*	*N/A*	(92)
			(93)
*Dlp1-KO, ZP121, and CHO*	*Increased peroxisomal abundance.*	*KD; Decreased peroxisomal abundance; peroxisomal elongation and aggregation.*	(93)
*COS-7*	*Increased mitochondrial fragmentation; perinuclear clustering; reduced viability; apoptosis; increased peroxisomal fission.*	*KD; increased mitochondrial elongation; perinuclear collapsed networks; peroxisomal elongation.*	(16, 17, 78, 94, 95)
*HAECs*	*N/A*	*Inhibited high glucose treatment-induced mitochondrial loss.*	(96)
*HEK293T*	*Increased mitochondrial fragmentation and intracellular calcium; No effect on mitophagy or apoptosis; Apoptosis after 30h.*	*KD; increased mitochondrial elongation; resistance to etoposide.*	(97, 98)
*HeLa*	*Collapsed networks; Increased mitochondrial fragmentation and perinuclear clustering; decreased cell viability with increased cytochrome c release; increased intracellular calcium; increased autophagy; increased apoptosis; no change in apoptosis or mitophagy; no change in mitochondrial fragmentation or Drp1 localization.*	*KD; increased mitochondrial elongation; perinuclear clustering; resistance to apoptotic stimuli.*	(16, 17, 19, 78, 79, 98–101)
*HCT116*	*Increased mitochondrial fragmentation and perinuclear clustering; mitochondrial recruitment of exogenous TBC1D15/17.*	*KD/KO; no effects on mitochondrial elongation; decreased Antimycin A-induced fragmentation; KO-cells had large LC3B punctae.*	(19, 102, 103)
*Human pancreatic β-cells*	*Increased mitochondrial fragmentation.*	*N/A*	(104)
*INS1*	*Increased mitochondrial fragmentation; decreased mitochondrial membrane potential; restored mitochondrial respiration in Fis1 depleted INS1 cells.*	*Increased mitochondrial elongation and size in glucose-responsive cells; resistance to STS; oxidized mitochondrial proteins; decreased ROS, mitophagy, mitochondrial respiration; no change in mitochondrial morphology but decreased Drp1 localization; prevention of high fat/high glucose induced mitochondrial fragmentation.*	(35, 104–106)
*Mouse embryonic fibroblasts (MEFs)*	*Increased mitochondrial fragmentation; slightly increased mitochondrial fragmentation in Drp1 KO background; increased apoptosis and Ca^2+^ retention; increased autophagy; unable to induce mitochondrial fragmentation in the absence of other OMM adaptor proteins.*	*Knockout increased mitochondrial elongation; decreased mitochondrial Drp1; decreased mitophagy; decreased OXPHOS-induced mitophagy; disrupted p62 mitochondrial localization; resistance to hypoxia, staurosporine, and etoposide.*	(20, 35, 99, 101, 107–110)
*Mouse cardiomyocytes*	*Increased mitochondrial fragmentation; apoptosis.*	*KD; increased mitochondrial elongation.*	(111)
*Primary human fibroblasts*	*N/A*	*KD; increased mitochondrial elongation and mass.*	(112)
*Primary mouse beta cells*	*N/A*	*No changes to mitochondrial morphology; inhibited high fat and glucose treatment-induced mitochondrial fragmentation.*	(105)
*Primary rat hippocampal neurons, and M17 cells*	*Increased mitochondrial fragmentation; perinuclear clustering; decreased neurite spine density.*	*Increased mitochondrial elongation.*	(113)
			(113)
*Rat cortical neurons (cultured)*	*Increased mitochondrial fragmentation; apoptosis.*	*N/A*	(114)

Despite these studies, Fis1’s role in Drp1-dependent mitochondrial fission is called into question by a number of discoveries that highlight the differences in the fission machinery between yeast and metazoans. The first inkling was the discovery that genetic ablation of Fis1 in *C. elegans* did not elongate mitochondria as expected (115). Similar results have been found in some mammalian cell lines in direct contrast to what has been found in yeast (19, 102). The discovery of three other Drp1 recruiters (mitochondrial fission factor aka Mff, MIEF1/2 aka Mid49/50) has reconciled some of these observations (19, 116, 117). Reconstitution of fission in budding yeast devoid of the native fission machinery shows that human Mff/Drp1 is functional, whereas human Fis1/Drp1 is not (21). Additionally, Mff and Mid proteins impact Drp1 hydrolysis, whereas Fis1 does not (21, 107, 118, 119). Collectively, these data have raised the question whether Fis1 is indeed involved in Drp1-dependent fission at all (103, 120, 121). Some clarification came with elegant mouse knockout studies in which mitochondrial morphology of Mff^-/-^, Fis1^-/-^, and Mff^-/-^/Fis1^-/-^ double knockouts were compared (20). Deletion of either gene is embryonic lethal but in mouse embryonic fibroblasts both Mff^-/-^ and Fis1^-/-^ show elongated mitochondria with a stronger effect upon deletion of Mff. The double knockouts (DKO) show an even more pronounced phenotype suggesting that Fis1 and Mff act independently of each other (20). Fis1 is not known to directly bind Mff, although earlier co-immunoprecipitation studies suggest that Fis1 may act downstream of Mff-mediated Drp1 recruitment and assembly at scission sites, however, the exact mechanisms remain unknown (102). Fis1 may act as a negative regulator of Mid51 recruitment of Drp1 as proposed (117). These and other observations noted below have led many in the field to propose that Fis1 is involved in a specialized fission that may be stress-induced, and Mff is the primary recruiter of Drp1 for fission involved in organelle distribution (19, 120). An open question in the field is to what extent the Drp1 recruiters cooperate with each other to effect membrane scission.

**Figure 3:**
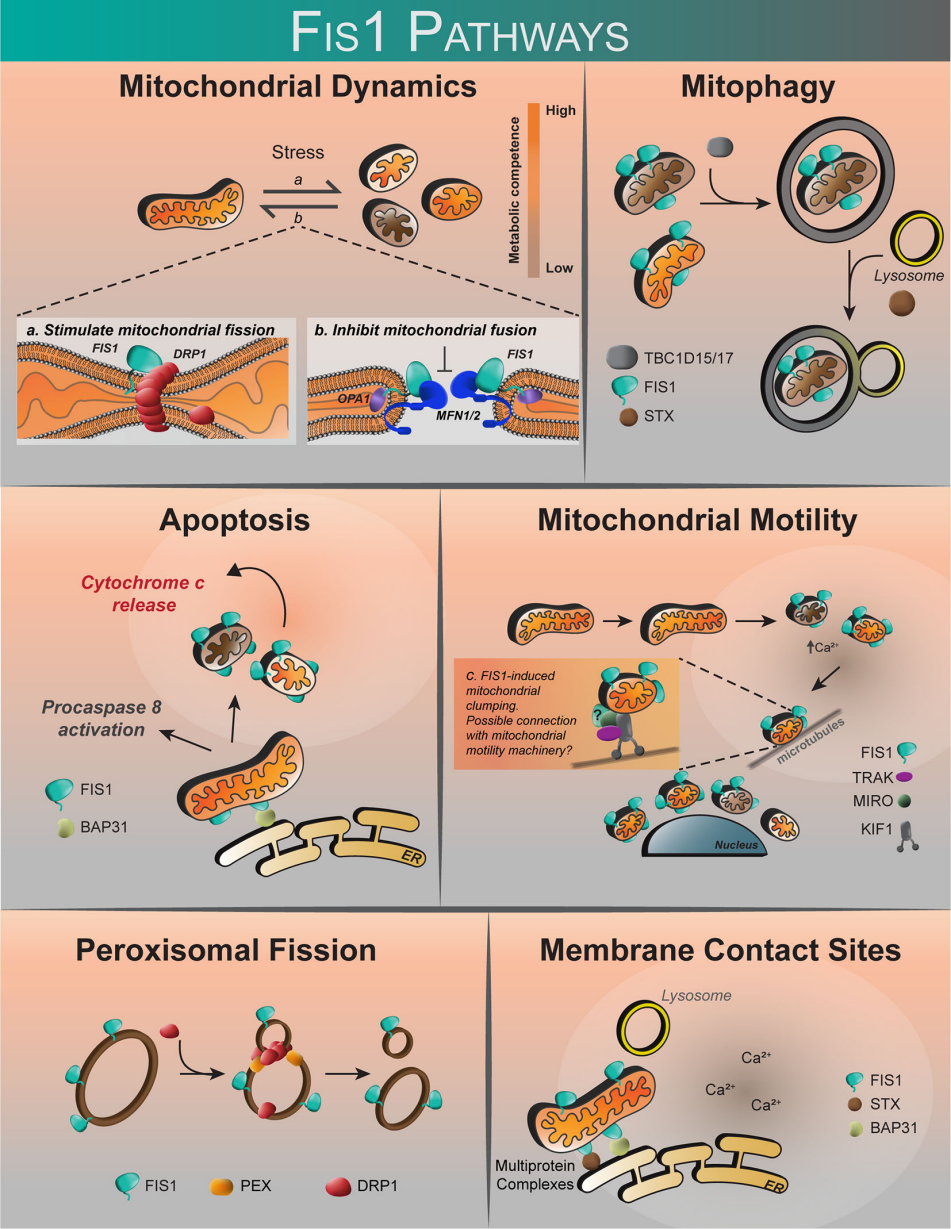
The proposed roles of human Fis1. Fis1 is proposed to participate in mitochondrial dynamics by stimulating mitochondrial fission *via* interactions with the mechanoenzyme Drp1, or by prevention of mitochondrial fusion through the inhibition of Mfn2/Opa1. Fis1 participates in mitophagy through recruitment of TBC1D15/17 and Syntaxin17 to mitochondria. Fis1 is also proposed to interact with BAP31, inciting apoptosis. Mitochondrial motility may occur through Fis1-induced mitochondrial clumping, or through interactions with the mitochondrial motility machinery consisting of TRAK, MIRO, and Kinesin. Fis1 has also been shown to participate in peroxisomal fission. It has also been suggested that Fis1 may be found at membrane contact sites, possibly in complex with BAP31 or Syntaxin17.

Contrary to a role in fission, Fis1 may cause fragmentation through an inhibition of fusion GTPases, Opa1 and Mfn1/2 (98). Interestingly, while Fis1 overexpression alone was sufficient to induce fragmentation and perinuclear vesicularization, the vesicular diameters were greater in the absence of Drp1, implying that Drp1 is still required for making smaller mitochondrial vesicles during fragmentation (98). Whether Fis1-Mfn interactions are cell type specific is not known, but might help explain previous work showing that Fis1-induced fragmentation required Drp1 (16, 17, 78). Complicating matters are discoveries that demonstrate an important role for Fis1 in mitophagy and apoptosis (**Figure 3**) that appear distinct from mitochondrial fission, yet still involve mitochondrial fragmentation (97, 99). In mitophagy, Fis1 recruits the proteins TBC1D15 and TBC1D17, which have GAP-like domains that act on Rab7 and 8, respectively, to limit autophagosome formation. Fis1 also interacts with Syntaxin17 in mitophagy during acute cellular stress (99, 103, 122). The role of Fis1 in mitophagy is discussed in greater detail below. Thus, the current body of literature (summarized in **Table 1**) does not provide a unifying picture for Fis1 function, which we interpret to arise from its multiple roles related to mitochondrial fragmentation that are likely tissue-specific; differences in experimental design and/or eukaryotic models may also contribute to apparent discrepancies. Nevertheless, it is well established that mitochondrial levels of Fis1 influence morphology (78), and high expression induces a distinct phenotype characterized by widespread mitochondrial fragmentation that eventually clusters around the nucleus. This perinuclear clustering of mitochondria can be accompanied by increased autophagy (78, 99, 101). Below we discuss the role of Fis1 in each of these proposed functions in more detail.

## Current Perspective on Human Fis1 in Mitochondrial Dynamics

### Mitochondrial Fusion and Fission: The “Kiss-and-Run” Model

Contrary to the popular idea that they act separately as functionally distinct machines, mounting evidence supports that mitochondrial fusion and fission can be closely coordinated in space and time. It has long been appreciated that the mitochondrial network morphology derives from a balance of fission and fusion (123, 124), however, the precise mechanisms underlying this balance are unknown. A “kiss-and-run” model characterized by alternating fusion and fission events is postulated (125) in which fusion can be either “complete” (mixing of integral membrane proteins) or “transient” (mixing of contents). Transient fusion events occur more frequently and were sufficient to maintain metabolic competence. Transient fusion may be explained by fusion and fission components physically juxtaposed at microdomains on ERMCs (30, 31), which suggests that mitochondrial networks can continuously execute bidirectional remodeling from a state of poise. Fis1’s newfound ability to inhibit the fusion machinery may be one way in which these processes are coordinated (98). The future goals are to determine the exact composition of ERMCs and the signals that govern them including integration with motility, mitophagy, and apoptosis that also reside at these contact sites.

### Mitochondrial Motility

Perinuclear clustering of mitochondria upon Fis1 overexpression implies that mitochondrial networks are effectively immobilized somehow, and may involve calcium signaling. Fis1 overexpression increases intracellular calcium by triggering a massive release of ER calcium stores (97, 108). Indeed, perinuclearly clustered mitochondria have been earlier shown to play important roles in regulating intracellular calcium (126). The mechanisms of mitochondrial motility are quite diverse since mitochondria can associate with cytoskeletal networks *via* multiple mechanisms [see here for an excellent review (127)]. However, mitochondrial motility is predominantly regulated by calcium concentrations as transient calcium fluxes are necessary for mobilizing mitochondria along cytoskeletal networks (128). Since mitochondrial motility is dependent on local calcium fluxes, Fis1 may influence the activity of the presiding machinery (**Figure 3**) by virtue of influence on intracellular calcium concentration.

Mitochondrial transport occurs on microtubule tracks by association with kinesin-1 (56, 57, 129). Miro and TRAK – which make up a functional complex together – are the only well characterized mitochondrially-resident proteins shown to govern mitochondrial mobility on cytoskeletal networks (130). Interestingly, Miro1, by virtue of its EF1-calcium sensing domain, was found to modulate mitochondrial shape transition (MiST) during GPCR-induced calcium stress in MEFs (131). In this mechanism, increased cytosolic calcium leads to a Miro1-mediated increase in the incidence of donut-shaped mitochondria. Although the MiST was not significantly perturbed in FIS1 and MFF DKO cells, their results suggest that increased cytosolic calcium acts upstream of Fis1/Drp1-induced mitochondrial effects. Since Fis1’s calcium flux effects are prominent at higher expression levels, it would be interesting to see how Miro1 activity is affected when Fis1 is highly expressed. At endogenous levels, Fis1 does not appear to exist in the same complex with Miro1, however, the temporal influence of ectopic Fis1 expression on Miro1 activity remains to be seen. Ectopic overexpression of either Fis1 or Miro1 is associated with increased intracellular calcium that is concomitant with upregulated mitophagy (55, 57, 101, 131, 132). In short, the mechanisms by which Fis1 modulates motility are not clear, however, its presence at ERMCs is notable given that calcium ions are dynamically transported between organelles (57, 133–136).

### Mitophagy

Mitophagy is a systemic culling of mitochondria by autophagy (137), and increases when cells are exposed to acute cytotoxic stressors (67, 138–141). A seminal finding was that mitochondrial fission events separated metabolically healthy from unhealthy daughter organelles with subsequent removal of the damaged daughter *via* mitophagy (35). Multiple mitochondrial proteins can mediate mitophagy depending on the physiological context, and excellent reviews can be found here (64, 65, 142–144). We focus on characterized roles for Fis1 in mitophagy (99, 102, 103, 122, 145).

Early evidence of Fis1’s involvement in mitophagy include initial findings that ectopic expression of Fis1 induced autophagy (101), and that mutations in Fis1 resulted in disorderly removal of autophagic vesicles (102). Interestingly, upregulation of Fis1 may have pathological roles in diabetes mellitus (96) and Parkinson’s disease (146) presumably through upregulating mitochondrial fission and mitophagy. Apart from a role in mediating mitochondrial fission directly upstream of mitophagy, emerging data from independent sources suggests that Fis1 plays other downstream roles in mitophagy (102). This is by virtue of Fis1’s influence on other proteins which are discussed in detail below.

#### TBC1D15 and TBC1D17

The discovery of the human Fis1 interactors, TBC1D15 and 17, was pivotal in shaping current beliefs that Fis1 plays specific roles in mitochondrial clearance by PARKIN-mediated mitophagy during conditions of acute cytotoxic stress (99, 103). In this proposed model, Fis1 helps to limit the size of growing autophagosomes at sites of mitophagy by recruiting TBC1D15/17 which, as GTPase activating proteins or GAPs, turns off the endosomal Rab7/8 GTPase activity. In support of this model, genetic ablation of Fis1 resulted in large LC3B puncta during mitophagy induced by PARKIN overexpression (103). These significant findings were largely dependent on ectopic overexpression as the Fis1-TBC1D15 interaction was undetectable at endogenous levels (99, 103). However, it may be that local concentrations are sufficiently high to promote this interaction at the ERMCs. It would be interesting to determine if Fis1-TBC1D15 complexes are actively present in this, or other, microdomains. Alternatively, Fis1 expression increases with various types of cell stress that might drive interaction with TBC1D15 during mitophagy.

In addition to stress mitigation, mitophagy also plays important roles in development and Fis1 may have functions there as well. For instance, recent work demonstrated that mitophagy is critical for the elimination of sperm-derived mitochondria post-fertilization (109). Strikingly, FIS1^-/-^ cultured mouse embryonic fibroblasts and pre-implantation embryos were unable to undergo successful mitophagy and removal of paternal mitochondria (109). Furthermore, a FIS1^-/-^ mouse is embryonic lethal, suggesting the protein likely performs other critical cellular functions during development (147). Indeed, there is growing evidence that Fis1 expression levels support cell pluripotency. In one mouse model, Fis1 levels could influence a pluripotent cell’s ability to differentiate through its influence on *De novo* fatty acid synthesis which required mitochondrial fission (148). However, minimal changes in Fis1 expression occur during mouse embryonic stem cell differentiation (149), suggesting further work needs to be done to elucidate the role of Fis1 during cellular differentiation.

Apart from PARKIN-mediated mitophagy, recent reports suggest that the Fis1-TBC1D15 interaction mediates interorganellar communication between mitochondria and lysosomes, which was reported to mark sites of mitochondrial fission and may allow for exchange of metabolites. Lysosomal untethering from mitochondria was dependent on the hydrolysis of Rab7-GTP, which was dependent on TBC1D15 recruitment by Fis1 (34). Interestingly, compared to wildtype, the rates of mitochondrial fission were markedly impaired when TBC1D15-GAP mutants or Rab7-GTP mutants were expressed. Likewise, inhibiting Fis1-TBC1D15 binding by expressing a leucine-alanine Fis1 variant (5LA) also reduced the rates of mitochondrial fission events. These recent findings are groundbreaking, especially since it suggests that the Fis1-TBC1D15/17 interaction may represent a novel means of fission independent of Drp1 activity (34, 99, 150).

Whether TBC protein recruitment is an evolutionarily conserved role of Fis1 is also an open question. TBC proteins have orthologs in yeast, but whether they interact with yeast Fis1p is unknown. Earlier studies in yeast showed that Fis1p was required for maintaining mitochondrial pools and evading apoptosis caused by ethanol treatment (151). Consistent with these results in yeast, deletion of Fis1p rendered cells more susceptible to yeast cell death induced by noxious hydrogen peroxide or acetic acid (152). Collectively, these findings might point to a primitive role for Fis1 in shaping mitochondrial dynamics, at least in times of acute mitochondrial stress.

#### Syntaxin17/STX17

Syntaxin17 (STX17) was initially identified as an autophagosomal SNARE protein required for fusing mature autophagosomes to lysosomes (153–155). Syntaxin17 is uniformly expressed on ER and mitochondrial membranes in fed cells, but concentrates at ER-mitochondria contact sites upon starvation. Syntaxin17 also exists in lipid raft-like domains which dynamically recruit Drp1 oligomers to mitochondrial sites of fission during fed conditions (156). Whether this Drp1 recruitment is Fis1-dependent is not known. In contrast, upon nutrient starvation a sub-population of Syntaxin17 congregates at ERMC sites where it presumably facilitates autophagosome-lysosome fusion during mitophagy (153, 154, 156–158). In support of this idea, STX17 ablation, or expression of a dominant negative variant, resulted in accumulation of undegraded autophagosomes even in fed conditions (159). Syntaxin17 was also identified to bind Fis1 which helps localize it at ERMC sites. Deletion of Fis1 causes increased mitophagy upon Syntaxin17 expression suggesting that Syntaxin17 activity is negatively regulated by Fis1. These data support that Fis1 might play early and late-stage roles in different forms of autophagy: an early role preventing Syntaxin17 induced mitophagy and a late role in limiting autophagosome formation. Strikingly, Fis1 mutants also show noticeable defects in autophagy as evidenced by the presence of large autophagosomes which are more apparent during acute mitochondrial stress (102).

The emerging FIS1-STX17 axis is particularly exciting as it directly links Fis1 to a primitive mechanism for mediating Drp1-dependent mitochondrial fission during conditions of stress (122, 158, 160). Since STX17 is present in most eukaryotes and absent in yeast (156), it is possible that it may have assumed some of the primitive functions of Mdv1p. Although evidence of this novel idea is sparse, Syntaxin17 bears some striking functional similarities to Mdv1p including an ability to recruit Drp1 to mitochondrial sites of scission, as well as influence mitochondrial dynamics in a Fis1-dependent manner. Other evidence includes structural considerations which indicate that Syntaxin17 contains a coil-coiled domain homologous to Mdv1p (87, 161). The coil-coiled moiety on Mdv1p mediates Fis1p-Dnm1p interaction at mitochondrial sites of scission. Interestingly, the N-terminal TPR domain of Fis1 – indispensable for Mdv1p interaction in yeast (83, 86, 87, 162) – is required for Syntaxin17 interaction (122). It is tempting to speculate that Syntaxin17 may play a Mdv1-like role in humans.

### Apoptosis

Mitochondria are critical for apoptosis and Drp1-dependent fragmentation accelerates this process (163, 164). Depending on the source of stimuli, canonical apoptosis can proceed *via* any of two distinct pathways – intrinsic or extrinsic – regulated by caspase proteases (165, 166). Although some degree of crosstalk exists (167–169), the intrinsic and extrinsic pathways are governed by effector caspases, CASP8/10 and CASP9 respectively (166). In the intrinsic pathway, intracellular cytotoxic cues trigger BAX/BAK-mediated mitochondrial-outer-membrane permeabilization (MOMP) (166, 170). MOMP signals a “point-of-no-return” in intrinsic apoptosis since it results in large scale efflux of mitochondrial proteins into the cytosol (171). Chief among these mitochondrial proteins is Cytochrome c since its cytosolic availability is a limiting step for apoptosome formation and activation of Caspase 9 (172). As a result, the mitochondria play a fundamental role in intrinsic apoptosis signaling. In contrast, the extrinsic pathway is initiated by cell surface receptors which cascade into death-inducing-signaling-complexes (DISC) required for processing and activating Caspase 8/10 (167–169, 173). With that in mind, apoptotic roles have been described for Fis1 in several experimental contexts (summarized in **Table 1**).

Fis1’s apoptotic functions are still rather unclear because it appears to be anti-apoptotic in some experimental settings, and pro-apoptotic in others. A possible explanation for this disparity is that Fis1 might be affecting multiple cellular processes – depending on the prevailing conditions – culminating as unique physiological outcomes. This idea is not particularly far-fetched since non-redundant roles for Fis1 in mitochondrial fission, mitophagy, and apoptosis have been described. Given that Fis1 probably has diverse cellular functions, another possibility is that its activity is dictated by expression thresholds. As seen in proteins such as kinases with pleiotropic functions, expression past certain thresholds can result in opposite physiological effects (174, 175). This notion is supported by transient overexpression experiments in which Fis1 improved mitochondrial connectivity at moderate levels, but enhanced fission and apoptosis at overexpressed levels (16, 17, 78). In the following sections, we summarize the experimental context regarding Fis1 functions during apoptosis.

#### Fis1’s Involvement in Intrinsic Apoptosis and MOMP

Fis1’s ability to trigger runaway mitochondrial fission and Cytochrome c leakage inexorably links its functions to intrinsic apoptosis (16, 17, 100, 176). However, this observation might be dependent on cell type and expression levels as Cytochrome c leakage is not always observed upon ectopic Fis1 expression (99, 102, 103). Also, overexpression of Fis1 potently induces massive recruitment of cytosolic Drp1 pools to mitochondrial sites (16, 78). Interestingly, even though it is not a required event (177–179), mitochondrial recruitment of Drp1 occurs upstream and may regulate BAX/BAK oligomerization in some cases (108, 179–181). While no direct evidence implicates Fis1 in BAX/BAK-mediated mitochondrial-outer-membrane permeabilization (MOMP), Fis1-induced mitochondrial dysfunction was abrogated in BAX/BAK-null cells suggesting a degree of cooperation (108). As stated earlier, MOMP is required for intrinsic apoptosis to occur, however, it is debated whether mitochondrial fission and MOMP are functionally tied. Mitochondrial fragmentation occurs at early stages of apoptosis (182), and its role in apoptosis has been heavily debated. Nevertheless, a prevailing consensus is that mitochondrial fission at least sensitizes cell death responses in a Cytochrome c dependent manner (179, 183–188). According to this model, unopposed mitochondrial fission effectively acts as a ‘primer’ for MOMP, perhaps by inducing membrane curvature or an initial leakage of Cytochrome c. This idea is supported by independent findings in which reducing levels of Fis1 protects cells from stress-induced apoptosis, whereas Fis1 overexpression enhances apoptotic response even under normal conditions (17, 100, 105). Since ectopic Fis1 expression often triggers cellular stress and apoptosis at very high levels (78, 97, 101, 108, 120, 176), it is not clear if Fis1 is playing any direct roles in intrinsic apoptosis. Thus, further investigation is needed to determine if Fis1 directly participates in MOMP and apoptosome formation.

#### Fis1’s Involvement in Extrinsic Apoptosis

Extrinsic apoptosis signaling begins on cell plasma membranes, like the intrinsic pathway, and also converges on mitochondria ultimately leading to MOMP as well (167, 168, 173). A role for Fis1 bridging intrinsic and extrinsic apoptotic signaling at ER-mitochondrial contact sites involves BAP31 (189), a resident ER molecule that exists in a ternary complex with procaspase 8 and Bcl-2 (190–192). BAP31 is preferentially processed by Caspase 8 into a truncated form, p20 BAP31, during early stages of apoptosis (193). Truncated BAP31 later was found to be pro-apoptotic because ectopic expression induced mitochondrial fragmentation and Cytochrome c release (186). In contrast, a caspase resistant BAP31 variant delayed apoptosis externally induced by the death-ligand, FasL, *via* preventing Cytochrome c release (194, 195). These results suggested that BAP31 cleavage linked extrinsic apoptosis with Cytochrome c release by unknown mechanisms.

One proposed mechanism is that Fis1 and BAP31 together make up a scaffold-like complex at ERMCs, dubbed the ARCosome, that is necessary for activating procaspase 8 (97). BAP31 cleavage was concomitant with widespread mitochondrial fragmentation which required Fis1. Strikingly, temporal expression of ectopic Fis1 had minimal effects on apoptosis and BAP31 cleavage until 30-48h post-transfection, suggesting an overexpression effect. This is also in line with findings where Fis1-induced Cytochrome c release was evident at 36 hours-post transfection (17). Nevertheless, the interaction between Fis1 and BAP31 was robust, even at endogenous levels of both proteins, supporting biological relevance (97). If this postulation holds, it may explain why Fis1-ablated cells are resistant to apoptosis inducers since Fis1 is required for ARCosome formation. Also, ER to mitochondrial calcium flux appears to play an important role here as shown earlier (108) since inhibiting ER calcium stores with thapsigargin diminished apoptotic responses. An independent report corroborated this idea by showing that BCL-2 co-overexpression rescued a pro-apoptotic variant of Fis1 (176). BCL-2 expression reduces intracellular calcium as well as inhibits mitochondrial calcium influx (196). This effect was not limited to BCL-2 because Fis1-induced cytochrome c release could also be partially rescued by chemical inhibition of mitochondrial calcium influx (176). These findings collectively support the initial idea that BAP31 may be acting upstream of BCL-2 and Cytochrome c release (197).

The Fis1-BAP31 model raises several interesting questions that include defining the ARCosome composition and whether Fis1 directly interacts with BAP31. It will be interesting to determine whether Fis1-induced Cytochrome c release is dependent on BAX/BAK-mediated MOMP, which might be fruitful to address in models of extrinsic apoptosis using recombinantly expressed death ligands (198, 199) that avoid inherent complications from triggering intrinsic apoptosis.

### Fis1 as a Double-Edged Sword in Apoptosis and Mitophagy

During conditions of acute cellular stress, both apoptotic and mitophagic pathways have the potential to be triggered (200). Depending on prevailing intracellular conditions, a tug-of-war between these pathways may ultimately determine cell fate. For instance, acute apoptotic stressors can trigger MOMP and sub-lethal activation of caspase-3 which could, in principle, be resolved by a robust mitophagic machinery thereby preventing cell death (201–203). Conversely, protease cleavage during apoptosis prevents autophagy in several examples including Beclin1/Vps34 (204), Atg5 (205), and AMBRA1 (206) suggesting tight coordination of these processes. Several molecules likely help orchestrate this cell fate decision including recent evidence that Parkin-dependent monoubiquitination of VDAC1 inhibits apoptosis, whereas Parkin-dependent polyubiquitination promotes mitophagy (207). A role for Fis1 in apoptosis and mitophagy suggests that it has both pro- and anti-apoptotic functions (97, 99, 101, 108, 122, 176) and might be similarly poised to be involved in the apoptosis/mitophagy decision. However, mechanisms that govern its regulation are not known. On one hand, Fis1 could help prevent widespread MOMP and apoptosis through mediating clearance of compromised mitochondria *via* mitophagy. Early evidence of this can be found in yeast models where Fis1p was required for survival during ethanol-induced apoptosis (151). On the other hand, Fis1 could mediate Caspase 8 activation and apoptosis in cases where cell damage/MOMP is too widespread and cannot be effectively contained by mitophagy (97). On a speculative note, the emerging FIS1-STX17-BAP31 axis may explain the functional switch in Fis1 activity since BAP31 is reported to inhibit Syntaxin17-induced mitophagy during stress (208). Interestingly, Fis1 also inhibits Syntaxin17-mediated mitophagy (122), suggesting that Syntaxin17 depletion and/or enrichment of BAP31 in this presumed complex is what necessitates a switch between Fis1-mediated mitophagy and apoptosis. In summary, we do not know what demarcates Fis1’s apparent pro- and anti-apoptotic functions but studies investigating its potential role as a switch between mitophagy and apoptosis may be fruitful.

## Extra-Mitochondrial Functions of Fis1

### Mitochondria-Associated Membranes

Fis1 is present in mitochondria associated membranes (MAM), but its extra-mitochondrial functions have yet to be elucidated (97, 122, 209). Characterized MAMs involving Fis1 include those between the mitochondria and the ER (also known as ERMCs), and those between mitochondria and lysosomes. Bidirectional remodeling of associated organelles may occur at these juxtaposed sites (150, 210–213). In addition to Ca^2+^ exchange, ER-mitochondrial contact sites are emerging as microdomains where lipids are dynamically exchanged between contacting organelles (210, 212, 214–216). At contact sites between mitochondria and lysosomes (34), untethering *via* TBC1D15/Rab7 was delayed upon mutating or depleting Fis1 (34). Whether Fis1 helps mediate an exchange of biomolecules between organelles independent of Drp1 or fission is an intriguing idea that would be consistent with membrane trafficking roles - during apoptosis and autophagy - of Fis1 interactors. Fis1 may also influence inter-organellar ion flux by influencing ER and lysosomal stores at MAMs. Specifically, calcium flux is intimately linked to mitochondrial dynamics/energetics and substantial evidence supports that Fis1 plays an important role. Excluding the ER, the mitochondria make up the largest intracellular calcium store within a cell (217, 218). Indeed, mitochondria act as slow, non-saturable calcium buffers in cases where ER buffering is compromised (217). Early evidence of Fis1’s involvement is found in reports where ectopic Fis1 expression was concomitant with massive release of ER calcium stores, as well as increased mitochondrial permeability to calcium (97, 108, 176). Interestingly, increased cytosolic calcium resulted in enhanced mitochondrial fission and autophagy by increasing Fis1 and Drp1 expression (219). These findings collectively suggest that a positive feedback loop exists between calcium signaling and Fis1/Drp1-mediated mitochondrial dynamics. Furthermore, a significant fraction of lysosomes also make stable contacts with mitochondria during normal homeostasis that may be important for inter-organelle calcium and ion flux (34, 150). These cations are important cofactors for key enzymes involved in mitochondrial bioenergetics, therefore, such contacts may serve as regulatory domains for lipid and ATP production (150, 210, 220–222). A non-fission role for Fis1 at MAMs may explain why FIS1-KO cells show deleterious perturbations to mitochondrial respiration (122), which could be a result of disrupted calcium signaling, lysosomal untethering, and/or autophagy. Conversely, ectopic expression of Fis1 does not interfere with mitochondrial respiration, unless at levels that trigger widespread fragmentation and BAX/BAK dependent Cytochrome c release (97, 108, 223). Collectively, the data imply a multifunctional nature of Fis1 that likely depends on expression levels or post-translational modifications, although no modifications have been reported. Therefore, in the future, it would be interesting to see how Fis1 expression/modifications correlates with the incidence of MAM sites, how this impacts exchange of biomolecules between organelles, and affects physiological outcomes.

### Peroxisomal Fission

Fis1’s role in peroxisomal dynamics is very similar to its role in mitochondrial dynamics in that its exact role is still actively debated. FIS1-KO cells do not show significant changes to peroxisomal morphology which is in stark contrast to MFF and DRP1-KO cells which had prominently elongated peroxisomes (19, 103, 224). These data indicate that Fis1 does not play limiting roles in peroxisomal dynamics. Nevertheless, ample evidence supports Fis1 involvement in peroxisomal fission (93, 94, 225–227). Most compelling of this evidence is the finding that siRNA-mediated silencing of Fis1 elongates peroxisomes, while ectopic expression increases the number of peroxisomes (93, 94). Interestingly, Fis1 was shown to interact with the peroxisomal biogenesis proteins, PEX11 and 19, and this interaction is required for Fis1 insertion into both mitochondria and peroxisomes (93, 226–229), although conflicting data exists (230). Since PEX19 apparently targets Fis1 to both mitochondria and peroxisomes, this implies a possible interplay between mitochondrial and peroxisomal membranes that may be dependent on Fis1 (226, 229). Indeed, a unique pathway has been discovered whereby mitochondrial derived vesicles (MDVs), which are generated in a Drp1-independent manner, actively traffic to peroxisomes (62, 231–233). MDVs are also targeted to other organelles apart from peroxisomes and what dictates MDV-peroxisomal trafficking is currently unclear (62, 233, 234). MDVs can be diverse and difficult to distinguish, and Fis1’s role in the process of MDV generation has not been studied directly (62, 235). However, MDVs are thought to be a stress-induced mitochondrial quality control mechanism, conditions under which Fis1 may be more important (62, 235, 236). Moreover, Fis1 appears to generate mitochondrial vesicles even in the absence of Drp1, although the average reported areas are much larger than the 70-150nm that is characteristic of MDVs (62, 98). Nevertheless, Fis1 may be modulating stress-induced MDV biogenesis through TBC1D15-mediated influences on RAB7 which is thought to be important for the process (62). This notion is plausible since mitochondrial recruitment of TBC1D15 and Rab7 is dynamic and minimal in healthy conditions, while mass recruitment of TBC1D15 to mitochondrial compartments is concomitant with Fis1-induced mitochondrial changes (99, 103). In addition, Fis1 interactor, Syntaxin17, is required for transporting PINK1/PARKIN-dependent MDVs to endolysosomes indicating that MDV generation and mitophagy are also closely linked (122, 157, 236).

In summary, the role of Fis1 in peroxisomal dynamics is controversial with evidence that it has active functions in stress-induced peroxisomal biogenesis and fission. Peroxisomes make up an integral part of cellular stress management, therefore, Fis1 may integrate molecular cues from both organelles in order to effectively manage homeostasis during stress, which warrants further investigation.

## Fis1 Dysregulation in Endocrine Diseases: Cause or Symptom?

Fis1 expression is frequently upregulated in a panoply of endocrine diseases; however, it is unclear if increased Fis1 is inherently pathogenic, or simply a symptom of the underlying pathology. Since Fis1 appears to negatively regulate a cell’s autophagic and apoptotic response in times of noxious stress, increases in Fis1 expression in the context of disease can be ambiguously interpreted. On one hand, increased Fis1 could be seen as a defense mechanism against transient spikes in noxious stress. Increased reactive oxygen species (ROS) can be a driver of mitochondrial fission, possibly facilitated through both transcription dependent and independent pathways (96, 237–242). Further, mitochondrial fission appears to be an inducer of increased ROS production, suggesting mitochondrial fission and ROS generation may occur in a feed-forward mechanism (243). The order in which these events occur, and if they occur in a cyclical manner, remains a major question in the mitochondrial dynamics field. Here we review major endocrine associated diseases in which Fis1 expression is markedly altered, while highlighting possible pathogenic roles.

### Diabetes

Diabetes is a group of metabolic disorders characterized by hyperglycemia secondary to impaired insulin response or secretion. This includes: type 1 diabetes (T1DM) which is due to total failure of insulin secretion secondary to autoimmune destruction of pancreatic beta cells; type 2 diabetes (T2DM) which is a multifactorial disorder characterized by resistance to insulin mediated glucose uptake, as well as a lack of compensatory insulin secretion; and gestational diabetes which is diagnosed by elevated blood glucose levels during pregnancy that may or may not resolve following delivery. Diabetes can also result as a complication of genetic variants leading to beta cell dysfunction or numerous diseases (244). T2DM is the most prevalent form world-wide, with approximately 1 in 11 individuals having been diagnosed with this disorder (245–247). Long term, T2DM is associated with complications affecting numerous organ systems including the kidneys, eyes, nervous system, and cardiovascular system. Chronic high glucose exposure damages the microvasculature, ultimately leading to abnormal perfusion and further complications (248, 249). In addition, high glucose levels can incite damage *via* other pathways such as hemodynamic alterations, osmotic changes secondary to sorbitol accumulation, and protein glycosylation (250). Diabetic patients are at an increased risk of death from all causes, with the highest rates of mortality attributed to myocardial infarctions and strokes, severe consequences of prolonged microvascular disease (251, 252). In addition to a predisposition to other health complications, diabetes is a significant driver of healthcare costs, with diabetic patients incurring approximately double the amount of medical care costs annually compared to those without diabetes (253).

The mechanisms underlying the pathogenesis of T2DM, as well as its numerous complications, are still unclear. However, a growing body of evidence suggests mitochondrial dysfunction (254), specifically abnormal mitochondrial dynamics, may play a role (96, 237, 255, 256). Mitochondria facilitate glucose stimulated insulin secretion in beta cells *via* the generation of ATP which activates a series of signaling events, ultimately leading to the opening of voltage-gated calcium channels and stimulation of insulin release (257, 258). In diabetic patients, this process goes awry due to mitochondrial dysfunction and a subsequent impact on the control of insulin secretion and response (259, 260). Islets from diabetic patients display decreased glucose-mediated insulin secretion, lower ATP levels, and impaired hyperpolarization of the mitochondrial membrane (261). Others have found that mitochondria from skeletal muscles of insulin-resistant diabetic patients are smaller in size with decreased bioenergetic capacities (262). Decreased mitochondrial size could be due to a decrease in normal mitochondrial fusion, or an increase in mitochondrial fission. Below, we focus on the contribution of Fis1 to the development of T2DM and T2DM related complications as the contribution of other mitochondrial dynamics proteins in these pathophysiologic mechanisms of T2DM have been well reviewed previously by others (238, 263).

### Insulin Resistance and Beta Cell Health

**Figure 4:**
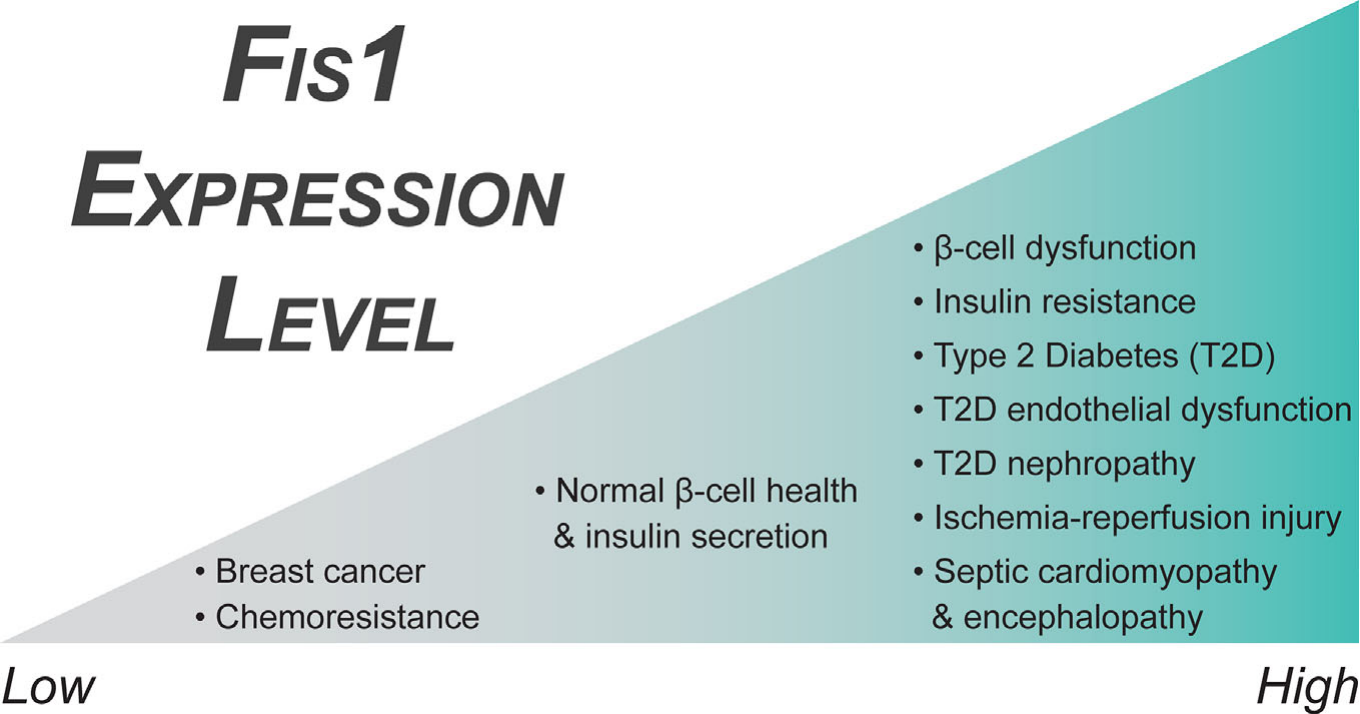
Fis1 expression in endocrine related diseases. Fis1 expression levels vary from abnormally low to high in different diseases, potentially impacting overall mitochondrial and cellular function.

Fis1 is a key regulator of pancreatic beta cell function, with precise protein levels needed for optimal glucose-stimulated insulin secretion (106). A minimum level of Fis1 protein is necessary for maintenance of the mitochondrial network and glucose responsiveness in beta cells (**Figure 4**), demonstrated by impaired mitochondrial dynamics and insulin secretion secondary to Fis1 knockdown (106). Similar impairment is observed upon Fis1 upregulation (**Figure 4**) and has been observed by others in glucose-responsive INS1 cell lines, as well as primary rat and human beta cells (104). These same processes are impaired in a glucose unresponsive cell line (INS1-832/2) which display elongated and clustered mitochondria, as well as decreased Fis1 expression and protein levels. This phenotype was improved upon overexpression of Fis1, leading to smaller mitochondria and glucose-stimulated insulin secretion (106). Fis1 also plays a prominent role in beta cell function in the setting of high fat, high glucose treatments (105). Prolonged beta cell exposure to elevated glucose and fatty acid levels is thought to contribute to beta cell dysfunction, impaired insulin secretion, and ultimately the development of type II diabetes (264). Exposure of INS1 cells to high fat, high glucose treatments led to mitochondrial fragmentation. Fis1 knockdown decreased fragmentation in these cells and inhibited recruitment of Drp1 to the mitochondria, an interesting observation that is congruent with Fis1 mediating Drp1 recruitment to the mitochondrial outer membrane. In addition, Fis1 knockdown led to decreased levels of apoptosis markers, including cleaved Caspase 3, DNA fragmentation, and Annexin V, suggesting Fis1 was involved in mediating cell death under these conditions (105).

Exposure to high levels of palmitate or myristate, two types of saturated fatty acids, in C2C12 muscle cells, as well as myocytes from the gastrocnemius of leptin deficient obese mice, induced both Fis1 and Drp1 upregulation and mitochondrial fragmentation. In addition, palmitate exposure decreased mitochondrial membrane potential and ATP production. Treatment with unsaturated fatty acids reversed mitochondrial fragmentation and functional deficits, and also stimulated insulin-mediated glucose uptake (265). The positive effects of unsaturated fatty acids on mitochondrial dynamics, specifically on Fis1 upregulation secondary to TNF-alpha mediated insulin resistance, has also been noted in adipocytes. Treatment with punicic acid prevented Fis1 upregulation induced by TNF-alpha, as well as improved cellular glucose uptake and both mitochondrial biogenesis and respiration. Further, punicic acid prevented ROS accumulation in the adipocytes (266). Others have also noted a possible role for Fis1 in cellular glucose uptake, exemplified by decreased GLUT1 and GLUT4 translocation to the plasma membrane upon Fis1 overexpression. These changes are accompanied by a concurrent downregulation of p-IRS1 and p-Akt, key regulators of insulin mediated responses (90). Although these researchers found no differences between high and low glucose conditions, others have found profound effects of high glucose exposure on mitochondrial dynamics and ROS production (239). It is possible that different cell types respond differently to high glucose treatment, and further work is needed to delineate the basis for these phenomena.

### Diabetic Cardiovascular Disease

Diabetic patients have a significantly increased risk of developing both microvascular and macrovascular disease compared to non-diabetic individuals, which increases cardiovascular morbidity and mortality (252, 267, 268). Hyperglycemic states, such as those in poorly controlled T2DM, are associated with hyper-fragmented mitochondria and increased Fis1 expression levels (**Figure 4**) (96). In addition, these vessels have increased production of reactive oxygen species that disrupts nitric oxide signaling decreasing normal vasodilation capabilities in the endothelium (96, 237, 239, 254, 269–271). Interestingly, Fis1 is over-expressed in human venous endothelial cells and Fis1 knockdown in cultured human aortic endothelial cells of diabetic patients ameliorates these effects (96). Diabetic endothelial dysfunction is known to precede the development of atherosclerosis and cardiovascular disease (272–274). As such, the most common cause of death in diabetic patients is myocardial infarction followed by other vascular complications (275, 276). Moreover, diabetic patients who experience a myocardial infarction and develop post ischemia-reperfusion (IR) injury do not respond to traditional cardioprotective therapeutics (277–279). This is a major clinical problem in T2DM where excess mitochondrial fragmentation – similar to endothelial dysfunction discussed earlier – is thought to be a major contributor to pathogenesis (237).

Murine cardiomyocytes, both *in vitro* and *in vivo*, that have experienced hypoxia-reperfusion conditions display upregulation of Fis1 (**Figure 4**) and Drp1, as well as a downregulation of fusion genes, in a casein kinase 2α (CK2α), a serine/threonine kinase, dependent manner (280). This alteration in mitochondrial dynamics genes results in excessive mitochondrial fragmentation. Further, CK2α disrupts normal mitochondrial dynamics by phosphorylating FUNDC1, an outer mitochondrial membrane protein that functions as a mitophagy receptor, and inhibiting FUNDC1-mediated mitophagy in a downstream manner, causing an accumulation of mitochondrial damage by reactive oxygen species (ROS), disruption of critical mitochondrial metabolic processes, and ultimately mitochondrial apoptosis (280). Fis1 and Drp1 upregulation, as well as mitochondrial fragmentation, has also been observed in both neonatal and adult cardiomyocytes secondary to increased cytosolic Ca^2+^, an early event in Ca^2+^ mediated ROS formation during IR injury (281). Similarly, overexpression of Fis1 in a human cell model of IR injury increases cell death, although it is unclear if this is truly an effect of IR injury, or merely due to Fis1 overexpression (16, 78, 282). Further supporting the idea of excess fission mediating IR injury in diabetes specifically, Drp1 inhibition is beneficial in a diabetic mouse model of IR injury (277, 283, 284). These interventions decrease infarct size, Troponin I levels, and ROS formation, as well as improve left ventricular dysfunction and cellular respiration *in vitro* and in rodent models, but have decreased efficacy in pig models of myocardial infarction (277, 282–286). These studies rely on the action of mdivi-1 (287), a putative Drp1 inhibitor with potentially profound benefits. However, the mechanism of action of mdivi-1 is controversial and includes inhibition of respiratory chain Complex I, even in Drp1^-/-^ cells (288–290). Although the precise mechanisms are not yet clear, the increased expression of Fis1 in conjunction with excess mitochondrial fragmentation in these models suggests Fis1 and mitochondrial fragmentation may play a role in the development of ischemia-reperfusion injury.

### Diabetic Nephropathy

Kidney disease is a common long-term complication of diabetes, affecting approximately 20-40% of all diabetic patients (291), and is a risk factor in the development of serious cardiovascular related complications (292). Despite the prevalence of renal complications in diabetic patients, the mechanisms governing the development of diabetic nephropathy are still poorly understood. Like diabetic endothelial dysfunction, mitochondrial hyper-fragmentation is observed in both *in vitro* and *in vivo* models of diabetic kidney disease. This hyper-fragmented phenotype is accompanied by metabolic dysfunction, increased ROS production, defects in mitophagy, and increased activation of apoptotic pathways (293–296). Several possible mechanisms of pathogenesis due to chronic high glucose exposure have been proposed, and disrupted mitochondrial dynamics leading to impaired mitochondrial function is a promising hypothesis. Chronic high glucose exposure, both in mouse models of diabetic nephropathy and human mesangial cell cultures, downregulates Mitogen-activated protein kinase phosphatase 1 (MKP1) (297), a protein previously suspected in the pathogenesis of diabetic nephropathy given MKP1 deficiency in the setting of renal oxidative stress causes tubular cell damage (298). MKP1 overexpression improved cellular metabolism and glucose control under hyperglycemic conditions, as well as a concurrent prevention of oxidative stress induced renal dysfunction, mitochondrial fragmentation, and apoptosis *via* inhibition of the Caspase 9 pathway.

The mechanism behind these improvements was found to be an inhibition of the JNK-CaMKII-Fis1 pathway as a result of the MKP1 overexpression (297). This suggests that high-glucose mediated downregulation of MKP1 allows for this signaling pathway to occur aberrantly. Direct effects of this included increased levels of both Fis1 (**Figure 4**) and Drp1 with a concurrent stimulation of mitochondrial fission. JNK signaling is an important mediator of cellular stress responses and apoptosis, including its role in insulin resistance and the development of type II diabetes, which has been reviewed extensively by others (299). A similar role of JNK signaling in pathologic Fis1 mediated mitochondrial fission has also been observed in tongue cancer as a function of Sirtuin 3 regulation (300). It does not appear that activation of JNK signaling guarantees Fis1 upregulation in all cases though, as JNK activation led only to increased Mff, not Fis1, in the setting of DUSP1 deficiency post IR (301).

High-glucose treatment of renal proximal tubular cells has also been associated with mitochondrial fragmentation from increased Fis1/Drp1 and decreased Mfn1, as well as increased levels of the redox and apoptosis regulating proteins Myo-inositol oxygenase (MIOX) and p66Shc (296, 302). These findings were corroborated in a later study using tissue samples from patients with diabetic nephropathy, and cellular work demonstrated improvement of the disrupted mitochondrial dynamics secondary to elimination of p66Shc (302). The authors also found evidence of a direct interaction between Fis1 and p66Shc, suggesting this protein may somehow be directly influencing mitochondrial fission. These effects were dependent upon p66Shc activation *via* phosphorylation of a serine at residue 36, which is believed to be performed by either JNK or protein kinase C (PKC) (303, 304). JNK was also determined to be a factor in the development of tubular cell damage under hyperglycemic condition *via* activation of the JNK-CaMKII-Fis1 in human mesangial cells (297). It is plausible that both groups have observed activation of the same pathways in different kidney derived models, but at different points of activation/signaling.

Interestingly, p66Shc knockdown did not alter Drp1 expression, suggesting that Fis1 may be the predominant downstream inciter of p66Shc driven fission during the development of diabetic nephropathy. P66Shc has been long implicated in vascular endothelial dysfunction (305, 306) and raises the question whether this pathway is also responsible for the mitochondrial fragmentation observed in diabetic endothelial dysfunction. A secondary role of p66Shc was also observed in this study in which p66Shc activation led to increased Mfn1-Bak interactions, ultimately increasing Bak activation, Cytochrome c release, and induction of apoptosis. It is not yet clear how the disruptions in mitochondrial dynamics interplay with Mfn1-Bak interactions, and if one of these processes occurs temporally before the other. However, the discovery of the Fis1-p66Shc interaction may help to link mitochondrial fragmentation with downstream activation of apoptotic pathways.

These discoveries raise the question of whether mitochondrial dynamics proteins disrupted in diabetic nephropathy can be targeted. Hispidulin, a flavone derivate found in numerous plant species with possible therapeutic benefits in cancer and oxidative stress, was able to counteract the negative effects of high glucose treatments in a murine podocyte cell model by inducing autophagy and inhibiting apoptosis (307). Specifically, hispidulin inhibited Pim1 and the regulation of the Pim1-p21-mTOR axis, ultimately resulting in decreased Fis1 expression, as well as decreased expression of the autophagy related proteins RAB18 and Park7. However, the resultant changes in protein expression appeared to be occurring through a decrease in mTOR levels, and not the typical mTOR phosphorylation regulatory pathway. As the mechanisms behind the development of diabetic nephropathy are elucidated, work towards a targeted therapeutic agent will become more achievable.

### BPDE and Fis1 Mediated Endocrine Dysfunction

Benzo(α)pyrene (BaP) is a well-established carcinogenic polycyclic aromatic hydrocarbon formed through incomplete combustion of organic substances. While low levels of polycyclic aromatic hydrocarbons are always present in the environment, some individuals are at risk of increased exposure through their occupations, individual environments, or lifestyle choices. High BaP exposure increases an individual’s risk of developing several different types of cancer, chronic diseases, and reproductive abnormalities (308). BaP exposure during pregnancy in a rat model results in decreased fetal survival, as well as decreased plasma concentrations of the critical gestational hormones progesterone, estrogen, and prolactin (309). One of the metabolic products of BaP is Benzo(a)pyren-7,8-dihydrodiol-9,10-epoxide (BPDE), an endocrine disruptor associated with trophoblastic dysfunction and disease. BPDE exposure increases Fis1 and Drp1 protein levels in Swan 71 human trophoblast cells. Mitochondrial fusion genes were also found to be downregulated, although it is unclear if this was a compensatory mechanism to the increased fission, or a direct result of the BPDE exposure. Increasing concentrations of BPDE induced ROS formation with a concurrent decrease in superoxide dismutase activity and decreased mitochondrial membrane potential. Cell death was induced *via* apoptosis with an upregulation of related proteins such as p53 and Bak1. Functionally, the cells exhibited decreased cellular invasion, a common trophoblastic process, as well as decreased human chorionic gonadotropin (hCG) hormone secretion which may explain the increased rates of miscarriages and growth restrictions during early pregnancies with BPDE exposure (310).

## Fis1 Expression in Oncogenesis and Cancer Progression

Mitochondrial dynamics plays an important role in cancer progression (311–314) and database searches show that normal Fis1 expression is frequently disrupted in cancers, however, it is not known how Fis1 expression impacts oncogenesis and/or cancer progression. Early evidence for potential roles of Fis1 in cancer can be gleaned from cardinal work using yeast genetics in which they showed that yeast Fis1 is genetically linked to a yeast specific stress-response gene WHI2 (315). Importantly, loss of Fis1 reduced respiratory competence that was independent of mitochondrial fission. Strikingly, Fis1 null cells consistently acquired cryptic WHI2 loss of function mutations which resulted in dysregulated growth control and apoptotic response. Their findings collectively imply that a loss of Fis1 can predispose cells to acquiring oncogenic traits. Consistent with this idea, the loss of Fis1 causes a severe loss of cell cycle progression at the G2/M checkpoint (316), consistent with other reports that Drp1 is involved in cell cycle regulation (317–319). Given this, it is possible that human Fis1 may play an evolutionarily conserved role in regulating cell growth. With this in mind, we will discuss two types of cancer, highlighting possible roles for Fis1.

### Ovarian Cancer

Ovarian cancer remains the most lethal gynecological cancer, partly due to the high rates of advanced stage disease upon diagnosis and relatively widespread chemoresistance to common platinum-based therapeutics (320). A small but growing body of evidence correlates disrupted mitochondrial dynamics to chemoresistance in cancers. Chemoresistant ovarian cancer cell lines show impaired apoptosis and hyperfused mitochondrial networks and inducing mitochondrial fission restores apoptotic capabilities and chemotherapeutic efficacy (321). It is possible that increased mitonetwork connectivity may be a way to promote cell survival by improving mitochondrial metabolic function (322). However, a later study found different expression patterns of mitochondrial fission proteins, with chemo resistant ovarian cancer cells displaying increased mitochondrial fragmentation (323). After evaluating tissue samples from 27 patients with serous ovarian carcinomas, a subset was identified with downregulation of the microRNA miR-488. When miR-488 was inhibited in a cellular system it increased cellular resistance to two common chemotherapeutic drugs, cisplatin and paclitaxel. In addition, these cells displayed a fragmented mitochondrial network. Conversely, treatment with a mimic of miR-488 resulted in decreased cell survival in the presence of the chemotherapeutics, as well as an elongated mitochondrial network. Of note, the mimic downregulated the mitochondrial fission proteins Fis1 and Drp1 and prevented phosphorylation of Drp1. It is unclear if the decrease in fission was simply due to changes in Drp1 level, or if the decrease in Fis1 also contributed *via* decreased Drp1 recruitment to the outer mitochondrial membrane. Fis1 downregulation as the result of microRNA has also been seen with miR-484 in both cardiomyocytes and adrenocortical cancer cells (111). Ultimately, these findings are in direct contrast to those discussed above, which may arise from different mechanisms of chemoresistance. Supporting this idea, miR-488 downregulation was only found in 11 of 27 patients, suggesting there may be multiple regulatory pathways in ovarian cancers converging on mitochondrial dynamics proteins. Ultimately, a better understanding of the role of mitochondrial dynamics in gynecological cancers, as well as in other cancers, may allow for more targeted therapeutics, especially in the case of chemoresistant cancers.

### Breast Cancer

Breast cancer remains the most common type of cancer in women, and one of the most prevalent malignancies worldwide (324). Given the prevalence and mortality, researchers have long been interested in understanding the mechanisms behind breast cancer pathogenesis and development of chemoresistance in the hope of designing better therapeutics. One of the greatest risk factors in the development of breast cancer is prolonged exposure to high levels of estrogen, a hormone with known mitochondrial activity. Despite its known roles in mitochondrial biogenesis, metabolism, and apoptosis, this hormone’s influence on mitochondrial dynamics is still developing (325–328). The effects of 17β-estradiol treatment on an estrogen sensitive MCF-7 breast cancer cell line down regulated Fis1 gene expression, but not Drp1, as well as increased expression of mitochondrial fusion proteins. Functionally, there were decreases in the protein levels of all mitochondrial respiratory chain components except for Complex V, as well as increase in ATP levels. Mitochondrial biogenesis also appeared to be stimulated, as expected, with increased levels of mtDNA (329).

A follow up study was performed in 2013 to determine the specific role of the estrogen receptors on their previous findings (330). In this study, the authors examined three breast cancer cell lines with different ERα/ERβ ratios, as well as a cell line with tetracycline induced silencing of the β receptor. They saw similar expression and functional patterns in the MCF-7 cells which had the highest ratio of β to α receptors out of the three cell lines tested. When the β receptor was then repressed in a different cell line, it recapitulated their findings, suggesting activation of the β receptor by 17β-estradiol is important in modulating mitochondrial dynamics and cell survival in some breast cancer cell lines. Fis1 has also been shown to be downregulated in tamoxifen resistant breast cancer cell lines as compared to chemosensitive parental cell lines, further suggesting downregulation of Fis1 (**Figure 4**) may contribute to mechanisms facilitating increased survival of malignant cells (331).

Expression of Fis1 also appears to be important in mediating radiation-induced mitotic catastrophe in a mouse breast cancer cell line, as well as innate response to oncogenic transformation (332). Ionizing radiation induces mitochondrial fragmentation *via* Drp1 dependent mitochondrial fission and knockdown of either Fis1 or Drp1 elongates mitochondria decreasing cellular radio-sensitivity (333). Further, intracellular calcium accumulation post radiation treatment, the proposed initiator of mitotic catastrophe, was largely suppressed (332). Similar observations have been made using knockdown of Fis1 or Drp1 in low and high dose radiation treatment of breast cancer (0.5 and 3 Gy respectively). Fis1 or Drp1 knockdown under high dose radiation improved cell survival, suggesting decreased cellular radio-sensitivity, whereas knockdown in the setting of low dose radiation appeared to actually promote apoptosis (334). Why these differences occur at varying levels of radiation is unclear but may suggest a critical point of damage upon which mitochondrial fission is necessary to promote radiation induced cell death. Fis1 upregulation has also been observed in whole blood samples from men undergoing radiation treatment for prostate cancer, possibly suggesting these Fis1 involved mechanisms regulating cell death post irradiation may be common to numerous tissue types (335). However, further research in more cell lines and patient derived samples are needed to determine this.

## The Role of Fis1 in Neuropathies and Neurodegeneration/Movement Disorders

Proteomics data suggest that at the protein level, Fis1 is ubiquitously expressed in virtually all healthy human tissues. Conversely, at the mRNA level, Fis1 is expressed in a wide range of levels across tissues. Notably, microarray datasets report Fis1 transcription to be significantly enriched in the heart compared to other organs, suggesting that Fis1 may have increased importance there. Also in line with an underlying tissue-specific importance, RNAseq datasets show the highest enrichment of Fis1 in the brain and skeletal muscle (336, 337). The variance in Fis1 mRNA expression in these tissues does not lead to a corresponding variance in protein expression, again buttressing a presumed “homeostatic demand” for similar amounts of Fis1 regardless of tissue type. The contrast between Fis1 mRNA and protein expression could also be due to varying rates of Fis1 elimination across tissue types. Fis1 is required for PARKIN-mediated mitophagy (**Figure 3**) but is also actively eliminated by the ubiquitin proteasomal pathway during this process (102, 103, 146). Therefore, a tissue’s homeostatic demand for Fis1 can, in theory, be governed by rates of mitophagy. Thus, one could reasonably infer that the highest demand for Fis1, and/or rates of mitophagy, occurs in the heart and brain. Consistent with this idea is the fact that Pink1 and PARKIN are highly expressed in both the heart and brain where they have important mitophagic functions crucial to cell survival (338–341).

**Figure 5:**
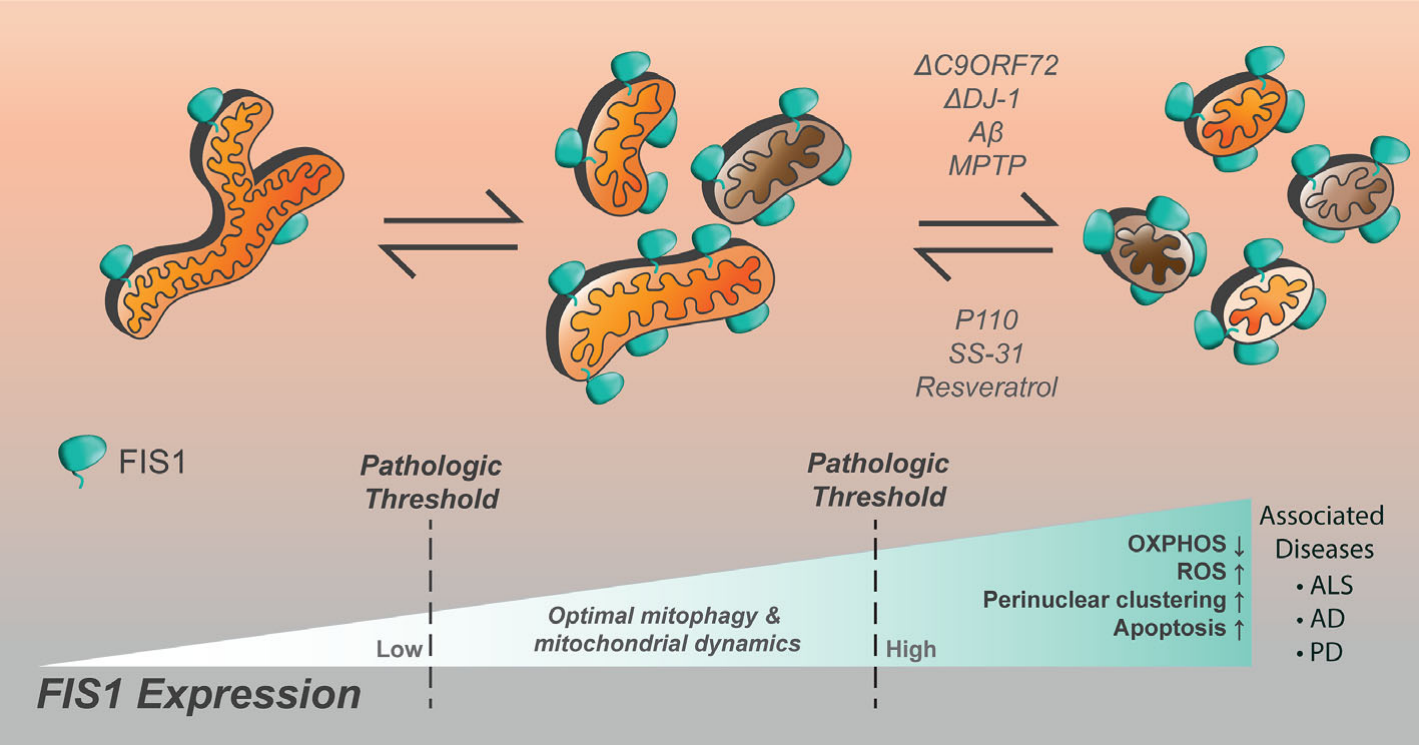
Increased Fis1 expression is noted in numerous neurological disorders, resulting in impaired cellular function. Mitochondrial fragmentation is worsened by loss of C9ORF72, DJ-1, the presence of amyloid-beta, or MPTP. The compounds p110, SS-31, and resveratrol have been noted to decrease mitochondrial fragmentation in the setting of Fis1 upregulation in models of neurological disease.

With regards to the brain, dysregulated mitochondrial dynamics and bioenergetics are among hallmarks associated with the progressive neuronal loss observed in Alzheimer’s (AD), Parkinson’s (PD), and amyotrophic lateral sclerosis (ALS), reviewed in (342–345). Since homeostatic demand for Fis1 appears to be highest in the brain, stress mediation by mitophagy is deemed crucial there. In line with this, evidence of dysregulated autophagy is often observed in the form of accumulated autophagic vesicles in AD, ALS, and PD (346). However, it is not clear if the observed vesicle accumulation signals an increase or inhibition of the autophagic machinery. Furthermore, genes frequently mutated in these three diseases are often mitophagy-related as seen with PINK1, PARKIN, DJ-1, and C9orf72. The molecular mechanisms of these diseases remain an active area of research, but evidence supports that mitochondrial dynamics are disrupted (347–349). Interestingly, Fis1 expression is markedly increased during the progression of all three neuropathies (**Figure 5**), however, it is not known whether it contributes to disease onset and/or progression. Current literature postulates that Fis1 has multiple molecular functions at MAMs in modulating both autophagy and apoptosis (16, 17, 34, 97, 99, 101, 103, 108, 122, 145, 176, 209). Thus an increase in Fis1 expression could, on one hand, signify a cellular crisis that is being mitigated by actively increasing Fis1-mediated mitophagy. On the other hand, Fis1 increase could occur passively due to inhibited autophagy and neuronal loss secondary to Fis1-induced apoptosis. Given this inherent ambiguity, Fis1’s involvement in the onset and progression of AD, ALS, and PD are discussed in depth below.

### Alzheimer’s Disease

One factor underlying AD progression is thought to be dysregulated mitochondrial energetics, concomitant with a fission-bias, which in turn compromises neuronal survival (342, 350–352). Multiple AD models report that Fis1 is upregulated as the disease progresses (113, 351). In one study, compared to normal controls, AD neurons had higher Fis1 levels, fewer neurite formations, and less mitochondria in formed neurites (113). Interestingly, while Fis1 overexpression could phenocopy AD-like mitochondrial distribution phenotypes in cultured neurons, Fis1 ablation failed to restore normal neurite formation even though mitochondrial connectivity improved (113). This suggests that increased Fis1 expression in the AD brain may be a response to AD pathogenesis as opposed to an inciter. Of note, transgenic mice models of AD do not differ in Fis1 expression (113, 353).

It is worth noting, however, that Drp1-mediated fission is postulated as one of the pathological culprits in AD due to its upregulation during disease progression (351). In support of this idea, AD-progression could be inhibited when Fis1-Drp1 interaction is presumably disrupted by peptide treatment (354). Curiously, Drp1 is not always upregulated during AD and in some models Drp1 expression can be reduced in response to AD-induction (355, 356). It is thought that temporal reduction in Drp1 serves as a possible compensatory mechanism to mitigate excessive mitochondrial fission (113, 355, 356). This is in line with electron microscopy results which also observed arrest of mitochondrial fission in AD neurons concurrently with reduced Drp1 activity (353). Although it is not clear what drives this excessive fission, ROS production is likely involved since treatment with the antioxidant peptide SS-31 could inhibit Aβ-induced mitochondrial fragmentation in a murine AD model (357). Furthermore, Aβ was found to upregulate Fis1, and to a lesser extent Drp1, the effects of which were attenuated upon SS-31 treatment. Similar results were seen upon treatment with resveratrol, a commonly prescribed antioxidant hypothesized to slow cognitive decline in AD patients (357). In summary, increased Fis1 expression and excessive fission is a phenotypic hallmark of AD and inhibiting Fis1-Drp1 mediated fission may have therapeutic benefits (**Figure 5**).

### Amyotrophic Lateral Sclerosis

Amyotrophic lateral sclerosis (ALS) is linked with mutations to genes with diverse cellular functions. However, regardless of the gene in question, chronic stress signals caused by ALS-inducing mutations generally coalesce on mitochondrial networks, ultimately disrupting their proper behavior and function (358, 359). Consistent with this notion, dysregulated mitochondrial energetics and trafficking is commonly observed in different models of ALS (360–364). Autophagy is also dysregulated in ALS and causal mutations to autophagy genes have been identified, key amongst which is C9ORF72 (**Figure 5**) (358, 365, 366). A hexanucleotide expansion repeat within C9orf72 is strongly linked to various forms of ALS and leads to the translation of poly-dipeptide repeat proteins (PDR) which have widespread deleterious effects on motor neuron survival (367–369). C9orf72 derived PDRs form insoluble p62-positive aggregates which inhibits normal nucleo-cytoplasmic shuttling while promoting stress granule formation (367, 370, 371). Consistent with this, ectopically expressed pathological C9orf72 bound to nucleoli and RNA-binding proteins, as well as induced cell death *in vitro* (372). C9orf72-induced aggregopathy is likely compounded by the loss of functional C9orf72 which can act as endosomal GEFs for Rab8A (373, 374), as well as initiate autophagy *via* Rab1/ULK1 (374–376). C9orf72 is also present on lysosomes where it was shown to modulate mTOR activity (377). Interestingly, Fis1 directly recruits both TBC1D15 and TBC1D17 which can act as endosomal GAPs for Rab7 and Rab8A, respectively. In addition, recruitment of TBC1D15 by Fis1 is important for untethering lysosomes from mitochondria at MAM sites. Taken together, these findings show that Fis1 and C9ORF72 both participate in the same functional axis.

In the case of ALS, autophagy is likely impeded leading one to infer that the observed Fis1 increase during ALS progression occurs passively (91, 362, 364). Thus, persistent Fis1 increase in ALS may very well adversely affect motor neuron survival by driving Drp1-mediated fragmentation and inducing apoptosis. Consistent with this idea is the observation that p110, a putative inhibitor of the Fis1-Drp1 interaction, was able to slow ALS progression in a murine SOD1 model (91). Apart from an inferred importance of Fis1 in ALS progression, a recent genome wide CRISPR-based screen showed that FIS1 is genetically linked to C9ORF72 (378). From this study, they found that genetic loss of both C9ORF72 and FIS1 resulted in synthetic lethality, implying that Fis1 and C9orf72 could functionally compensate for each other. Importantly, this study demonstrated that completely ablating Fis1 would likely be deleterious even in ALS-prone situations. To summarize, impeded proteostasis is likely involved in causing the increased Fis1 expression observed in ALS models, and specifically targeting Fis1’s fission function, possibly by inhibiting the Fis1-Drp1 interaction, has shown some promise in drug discovery.

### Parkinson’s Disease

Genetic interactions governing the etiology of hereditary PD perhaps offers the strongest evidence yet that increased Fis1 levels may be involved in the progression of neurodegenerative diseases. PD progression is mainly characterized by a cumulative loss of dopaminergic neurons within the substantia nigra (379). Classical loss of function mutations on three main genes – PINK1, PARKIN and DJ-1 – are strongly associated with autosomal recessive forms of PD (AR-PD) (380). Strikingly, these genes all have strong functional links to both Fis1’s expression and function in the context of stress mitigation by mitophagy.

AR-PD associated mutations in PINK and PARKIN were identified early by clinical studies, but their functional link was unknown (381). Attempts to model PD progression by ablating PINK1 or PARKIN have largely failed to completely recapitulate the phenotypes associated with the disease (381), suggesting that polygenic interactions are likely involved in autosomal recessive forms of PD. Functional links between PINK1 and PARKIN showed that they are both important mediators of stress-induced mitophagy, a process in which Fis1 may play a regulatory role (103, 145, 382–384). Downstream of PARKIN-mediated mitophagy, Fis1 recruits endosomal GAPs (TBC1D15/17) to sites of scission where they inhibit Rab7 activity thereby limiting autophagosome size (99, 103). It is not completely understood how clinical PINK1 or PARKIN variants associated with PD influence mitophagy, but evidence suggests they dysregulate mitochondrial dynamics especially during stressful conditions (67, 382, 385, 386).

Depletion of outer-membrane proteins on the mitochondria (by OMMAD) is concomitant with mitophagy (381). Fis1 is actively degraded during mitophagy, perhaps, *via* the proteasomal pathway (148). A murine model of Parkinsonism demonstrated that the E3 ligase, RNF5, ubiquinates Fis1 downstream of DJ-1 loss, and is required for Fis1 degradation (146). Findings from this model largely support a secondary role for increased Fis1 in PD progression, at least in the case of DJ-1 mutations. In these examples, loss of DJ-1 results in increased Fis1 expression due to impaired proteostasis and this increase is presumably deleterious (**Figure 5**). This is plausible as increased Fis1 levels induce perinuclearly clustered, fragmented mitochondrial networks that are also associated with murine PD phenotypes (288, 381). As such, increased Fis1 may be upstream of dopaminergic neuron loss in the substantia nigra given increased Fis1 is known to be pro-apoptotic (97, 108, 176). In line with this notion, the peptide p110, a putative inhibitor of the Fis1-Drp1 interaction, had neuroprotective effects in a 1-methyl-4-phenyl-1,2,3,6-tetrahydropyridine (MPTP) murine PD model (181).

## Fis1 in Therapeutic Advancements and Drug Discovery

Based on current literature, Fis1 appears to have a myriad of functions related to mitochondria and associated membranes, in addition to possible pathological roles arising from its dysregulation. To date, most efforts have focused on targeting pathological fission by targeting Drp1. Specifically targeting the Fis1-Drp1 axis is conceptually sound given Fis1 expression is frequently increased and its deleterious effects can be attributed to endogenous Drp1 activity; however, Fis1 inhibition of fusion cannot be ruled out. Increases in Drp1 expression are frequently associated with increased mitochondrial fission, however, ectopic expression of Drp1 does not increase fission *in vitro* (12) except at extremely high levels (387). Rather, it is the enrichment of Drp1 activators or recruiters like Fis1 and Mff on mitochondria that necessitates increased fission (387). Also, mounting evidence suggests that Fis1 may play a more dominant role than Mff in stress-mediated and pathological fission. Furthermore, at this time, it is apparent that a major disruption to basal intracellular stress signals is thematic of increased Fis1 expression. Indeed, peptides designed to inhibit Fis1-Drp1 and Mff-Drp1 suggest that targeting Fis1 has more therapeutic benefits, at least in neurodegenerative models. Perhaps the most compelling physiological evidence yet that depleting Fis1 has a therapeutic benefit in a pathological context is in the diabetic endothelium, where Fis1 expression and mitochondrial fragmentation is increased (96). A pathological hallmark of DM physiologically presents as endothelial cell dysfunction resulting in more than a 30% decline in vasodilatory responses to acetylcholine (Ach). In endothelial cells obtained from DM patients, increased expression of Fis1 and Drp1 coincides with highly fragmented mitochondrial networks. Further, activation of the endothelium-derived NO synthase was improved in human aortic endothelial cells exposed to high glucose and transfected with Fis1 siRNA to block Fis1 over-expression (96). These data suggest that Fis1 levels, at least in the diabetic endothelium, may have crossed a pathological threshold. Further, these results suggest that Fis1 inhibition/depletion in such pathological contexts may not only have inhibitory effects on disease progression but may also actively reverse phenotypes in some cases.

Targeting and inhibiting proteins involved in mitochondrial dynamics is of growing interest. Specifically, mitochondrial fragmentation is a characteristic finding in numerous pathological processes, and thus, finding a way to inhibit excess mitochondrial fission may improve patient outcomes. Currently, two of the most well studied fission inhibiting compounds are mdivi1 and p110. Mdivi1 (mitochondrial division inhibitor 1) is a quinazolinone derivative discovered *via* a chemical screen in yeast and is a proposed Drp1 inhibitor (287); whereas p110, a small seven-residue peptide derived from a homology sequence between Fis1 and Drp1, is thought to disrupt the Fis1-Drp1 interaction in a specific manner (288). It remains questionable though if mdivi1 is truly Drp1 specific as it only mildly inhibits Drp1 GTPase activity (*K*_i_ > 1mM), is a reversible complex I inhibitor, and modifies mitochondrial ROS production (289). Further, mdivi1 is capable of regulating outer mitochondrial membrane permeabilization to block Bak/Bax dependent Cytochrome c release, and these functions may instead contribute to its beneficial effects (287). Further, as a quinazolinone it likely has many cellular targets. Regardless of its mechanism of action, mdivi1 is certainly an interesting molecule whose properties have been shown to be beneficial in various cell based models of disease.

Similarly, p110 was designed to inhibit Drp1 and clearly possesses cellular activity that is consistent with this; however, no *K_i_* has been reported and studies interrogating whether it directly inhibits Fis1-Drp1 would be welcome. Regardless, given the proposed role of Fis1 in numerous diseases, p110 may be particularly beneficial in targeting numerous pathological processes. Many of the studies currently published that utilize these compounds are in the context of targeting ischemia-reperfusion injury, typically with significant benefits in mitochondrial morphology and function both *in vitro* and *in vivo* (277, 282–285, 290, 388). There have also been studies examining the use of p110 and thus the proposed disruption of the Fis1-Drp1 interaction in models of sepsis related complications such as cardiomyopathy and encephalopathy (389), as well as neurological diseases including Huntington’s disease, amyotrophic lateral sclerosis, and Parkinson’s disease; of which, these studies found significant improvement in pathogenesis and downstream effects of the diseases (91, 354, 390–392). These improvements are determined by criteria such as inhibition of excess mitochondrial fission, decreased reactive oxygen species production, improved mitochondrial membrane potential, and decreased apoptosis. Functional improvements of both the cells and model organisms have also been noted. Of note, minimal effects of p110 on basal mitochondrial fission in neurons were found (288), an important finding given some amount of mitochondrial fission is required for cell survival and homeostatic responses (35, 393–395).

The use of p110 in pulmonary arterial hypertension has also been evaluated with mixed conclusions. Administration of both p110 and mdivi1 improved mitochondrial structure, membrane potential, and function, as well as right ventricular diastolic function in a rat model of pulmonary arterial hypertension (396). However, a follow up study showed that p110 was only capable of reducing mitochondrial fission and proliferation of right ventricle fibroblasts at very high doses (397). In addition, p110 was unable to prevent or improve any right ventricular fibrosis or dysfunction *in vivo*. Ultimately, although excess mitochondrial fragmentation is noted in pulmonary arterial hypertension, the role of Fis1 in this process is still debated (398, 399).

A second peptide SS-31, known to be beneficial in numerous ROS associated disorders such as Alzheimer’s disease (357, 400), the development of type 2 diabetes related cardiovascular disease (401), and sepsis-associated encephalopathy (402), also appears to affect mitochondrial dynamics. Interestingly, all of these disorders have been linked to disrupted mitochondrial dynamics and Fis1 upregulation as described throughout this review. The peptide is a cell-permeable ROS scavenging peptide that targets and is thought to accumulate in the inner mitochondrial membrane (403–405). Beneficial effects of SS-31 may also be due to a down-regulation of Fis1 and attenuation of excess mitochondrial fragmentation (406). In this study, the authors showed microglial cells upregulated Fis1, iNOS and Cox2, as well as exhibited more fragmented mitochondrial networks upon exposure to lipopolysaccharide (LPS). These detrimental findings were attenuated upon treatment with SS-31. Similar results were seen with a significant downregulation of Fis1, shown to be upregulated secondary to Aβ treatment, upon SS-31 treatment in mouse neuroblastoma cells used as a model of Alzheimer’s disease (357). It is not surprising that SS-31 was shown to be beneficial in a sepsis-associated encephalopathy mouse model given this process is also driven by the microbial endotoxin. It does however raise the question of whether the beneficial effects seen are also due to suppression of Fis1, or from the stabilization of cristae by aggregation of cardiolipin (404).

While the concept of inhibiting Fis1 mediated mitochondrial fission is promising, additional work clearly needs to be done in additional cellular systems and models of diseases in which Fis1 appears to be implicated. It is possible that a Fis1 inhibitor, as opposed to a Drp1 inhibitor, would be more specific for pathological fission given Fis1 appears less critical for normal homeostatic fission (101, 102, 110, 237, 407). However, Fis1 also appears to have other functional roles in mitochondrial motility, mitophagy *via* its proposed interactions with TBC1D15, TBC1D17, and Syntaxin 17, intrinsic and extrinsic apoptotic pathways, peroxisomal fission, and ER-mitochondrial contact points. It is possible that Fis1 inhibition would therefore be subject to numerous detrimental off-target effects, many of which directly influence a cell’s viability. Ideally, a Fis1 fission-specific inhibitor would only bind to and disrupt the Drp1 interface, while still allowing for Fis1 to interact with its other native ligands. To achieve this though, a better understanding of the role of Fis1 in mitochondrial fission, from a mechanistic and structural biology standpoint must first be achieved.

## Discussion—Outstanding Questions and Future Directions

As highlighted here, Fis1 appears to be an important molecule in key cellular processes. Many outstanding questions need to be answered in order to better understand the many potential activities of Fis1 in normal and pathological situations. As a TPR containing protein, Fis1 may interact with many other protein partners, and defining these partners would be a significant advance to the field. Towards this goal, proximity labeling of Fis1 identified TBC1D15 as the top hit, and statistically significant hits with gelsolin, tropomyosin, and spectrin (107) suggest that Fis1 plays a still undefined role in mitochondrial motility *via* cytoskeletal interactions. Presumably, other cognate binding partners of Fis1 exist and what these proteins are, and what governs their interactions are important outstanding questions. As noted above, Fis1 expression levels alone appear to drive some protein-protein interactions, such as with Drp1. Pathologically high Fis1 expression raises the question of what the fundamental roles of Fis1 at endogenous or sub-pathological levels are, which could include responding to intracellular changes that require attenuation of mitochondrial function through changes in dynamics. The finding that extrinsic apoptosis signals through Fis1-Bap31 interactions at endogenous levels raises the question of what other extracellular signal pathways converge on Fis1 for control of mitochondrial homeostasis. Given that Fis1 appears at a nexus of homeostatic, mitophagic, and apoptotic fission, determining these pathways is a high priority. The known interactions with the Tre2/Bub2/Cdc16 Domain Family Member 15 (TBC1D15) might offer a clue. TBC1D15 acts as a GTPase Activating Protein for at least one Rab protein, Rab7 (408, 409). Given that Rab proteins can mediate endolysosomal signaling (410), as well as mitochondrial division (34, 411), it is intriguing to speculate that perhaps the FIS1/TBC1/RAB axis might be involved in extracellular-to-mitochondria sensing.

Another major question is whether Fis1 overexpression observed in pathological conditions is causative or correlative. From the recent work in diabetes, it appears to be a driver of poor vascular vasodilation, but work needs to be done to fully address this outstanding question and determine its role in other diseases. In this regard, a major question is what occurs first: mitochondrial derived ROS that stimulates mitochondrial fragmentation or vice-versa? Certainly, mtROS generating conditions induce fragmentation, but it also appears that fragmentation induces ROS formation. Likely a feed forward loop exists, but dissection of this relationship may help resolve the causation/correlation question. Finally, it will be interesting to see whether targeting Fis1 pharmacologically will help resolve these questions and lead to the therapeutic promise shown by other modulators of mitochondrial dynamics.

## Author Contributions

UI and KM conceptualized, wrote, and edited the manuscript. KM designed the figures with input from all authors and the aid of MH. BH oversaw the review layout and writing, as well as provided feedback and editing of the manuscript. MW edited and provided feedback throughout the writing process. UI performed final edits and formatting. All authors contributed to the article and approved the submitted version.

## Funding

The authors are supported in part by grants from the National Institutes of Health (BH: R01GM067180, MW: R01HL128240, KM: TL1TR001437). The content is solely the responsibility of the author(s) and does not necessarily represent the official views of the NIH.

## Conflict of Interest

The authors declare that the research was conducted in the absence of any commercial or financial relationships that could be construed as a potential conflict of interest.
